# Ultrasonographic Signs of Cytomegalovirus Infection in the Fetus—A Systematic Review of the Literature

**DOI:** 10.3390/diagnostics13142397

**Published:** 2023-07-18

**Authors:** Magda Rybak-Krzyszkowska, Joanna Górecka, Hubert Huras, Magdalena Staśkiewicz, Adrianna Kondracka, Jakub Staniczek, Wojciech Górczewski, Dariusz Borowski, Mariusz Grzesiak, Waldemar Krzeszowski, Magdalena Massalska-Wolska, Renata Jaczyńska

**Affiliations:** 1 Department of Obstetrics and Perinatology, University Hospital, 30-551 Krakow, Poland; jsienicka@gmail.com (J.G.); huberthuras@wp.pl (H.H.); staskiewicz.ma@gmail.com (M.S.); 2 Hi-Gen Centrum Medyczne, 30-552 Krakow, Poland; 3 Department of Obstetrics and Pathology of Pregnancy, Medical University of Lublin, 20-081 Lublin, Poland; adriannakondracka@wp.pl; 4 Department of Gynecology, Obstetrics and Gynecologic Oncology, Medical University of Silesia, 40-055 Katowice, Poland; jstaniczek@sum.edu.pl; 5 Obstetrics and Gynecology Ward, Independent Public Healthcare Institution in Bochnia, The Blessed Marta Wiecka District Hospital, 32-700 Bochnia, Poland; wojtekg1_9@op.pl; 6 Provincial Combined Hospital in Kielce, Clinic of Obstetrics and Gynaecology, 25-736 Kielce, Poland; darekborowski@gmail.com; 7 Department of Perinatology, Obstetrics and Gynecology, Polish Mother’s Memorial Hospital-Research Institute in Lodz, 93-338 Lodz, Poland; mariusz.grzesiak@gmail.com (M.G.); waldemar.krzeszowski@gmail.com (W.K.); 8 Department of Obstetrics and Gynecology, Medical University of Lodz, 93-338 Lodz, Poland; 9 Salve Medica, 91-210 Lodz, Poland; 10 Clinical Department of Gynecological Endocrinology and Gynecology, University Hospital, 30-551 Krakow, Poland; mmassalska@su.krakow.pl; 11 Department of Obstetrics, Perinatology and Gynecology, Medical University of Warsaw, 02-091 Warsaw, Poland; renatajaczynska@tlen.pl

**Keywords:** cytomegalovirus (CMV), ultrasonography, fetal neurosonography, immuno assays, real-time PCR

## Abstract

Background: congenital cytomegalovirus (cCMV) infection during pregnancy is a significant risk factor for fetal and neonatal morbidity and mortality. CMV detection is based on the traditional ultrasound (US) and MRI (magnetic resonance) approach. Methods: the present review used the PRISMA protocol for identification of studies associated with CMV infection and sonographic analysis. Various search terms were created using keywords which were used to identify references from Medline, Pubmed, PsycInfo, Scopus and Web of Science. Results: sonographic analysis of the cCMV infection identified several of the key features associated with fetuses. The presence of abnormal patterns of periventricular echogenicity, ventriculomegaly and intraparenchymal calcifications is indicative of CMV infection in the fetus. Hyperechogenic bowels were seen frequently. These results correlate well with MRI data, especially when targeted transvaginal fetal neurosonography was carried out. Conclusions: ultrasonography is a reliable indicator of fetal anomalies, due to cCMV. Fetal brain and organ changes are conclusive indications of infection, but many of the ultrasonographic signs of fetal abnormality could be due to any viral infections; thus, further research is needed to demarcate CMV infection from others, based on the ultrasonographic approach. CMV infection should always be an indication for targeted fetal neurosonography, optimally by the transvaginal approach.

## 1. Introduction

Cytomegalovirus (CMV) is a member of the Herpesviridae family. The virus is spread through blood-borne transfer or by blood transfusion or transplantation. CMV infection is quite ubiquitous. In low- and middle-income economies it affects approximately all of the population by early childhood, and about half of the population by adulthood in high-income countries [1]. High-risk populations are susceptible to developing the CMV illness, including those who are immunologically compromised or vulnerable organ transplant recipients. Additionally, CMV is a significant contributor to morbidity and sporadic neonatal mortality [2]. CMV is one of the major causes of congenital infections during pregnancy. It possesses a significant risk factor during pregnancy. Approximately 4% of the babies die in utero or shortly after birth due to CMV infection. Additionally, it is the most frequent reason for cognitive defects [3,4]. Epidemiological data on CMV infection and subsequent antenatal outcomes in Israel showed that these infections are widely prevalent, with loss of pregnancy being associated with CMV transmission to fetus [5]. CMV is the main infection-related cause of congenital malformations in high-income countries, the most important non-genetic contribution to sensorineural hearing loss (SNHL), and a considerable contributor to neurological disabilities. Up to 10% of all cases of cerebral palsy and 7.9–20.9% of all congenital SNHL can be attributed to it at childbirth; by the age of 4 years old, this jumps to 25%, due to late-onset hearing impairment [6,7]. Due to the great diversity of the human cytomegalovirus (HCMV) genome and the lack of effective anti-infection strategies, both reinfection and the resuscitation of the endogen quiescent strain are possible [8]. The virus can be passed on directly or indirectly by contact with urine, cervical, oropharyngeal, and vaginal secretions, semen, blood products, or organ transplants. The virus sheds for a very long time after the first infection. Seroprevalence increases with age and is higher in individuals with lower socioeconomic status, both in high- and low–middle-income countries [9]. In the United States and Western Europe, seroprevalence spans from 50% to 85%, with the incidence of CMV primary infection during pregnancy between one and two percent. Primary infections during pregnancy are more likely in women who are young and have at least one child. In the United States, there was a 5.9% yearly chance of primary infection in pregnant women who had tested negative for the virus in their prior pregnancy. A recent French study found that compared to the general population, women who were seronegative at the time of their first pregnancy and who became pregnant within two years had a 19-fold greater risk of primary fetal infection during the first trimester and a 5-fold greater risk of side effects developing in their child [10]. CMV seroprevalence varies by geographical region; it is similar in the US and Western Europe, but significantly higher in Brazil and India Undefined maternal nonprimary infection incidence was 10% annually in young women in a three-year research study conducted in the United States. It is not yet clear if late-onset SNHL occurs due to the reactivation of the virus or due to immunological host response. In fetuses, injuries of the inner ear, specifically the cochlea, are diffuse and comprise both inflammation and cytomegalic cells with inclusion bodies [10,11,12,13,14,15,16,17,18,19].

Given that up to 90% of infections in pregnant women are undetectable and that the indicators that are available are non-specific, it is challenging and inaccurate to diagnose primary CMV in pregnant women based exclusively on clinical manifestations (arthralgia, myalgia, headache, fatigue). Pregnancy does not appear to have an effect on clinical severity. Serology is the only way to tell if someone has primary CMV infection, and seroconversion is demonstrated when CMV-specific IgG is found in a pregnant woman’s serum after she previously tested IgG-negative in a serum sample collected earlier in her pregnancy. Only when maternal blood samples are kept, as is typical in several countries, or when a screening test is in place and seronegative women are identified and closely monitored, can this technique be used [20,21]. Running a serology test that detects both IgM and IgG levels is the accepted standard way of identifying primary CMV infection in the complete absence of seroconversion and based on clinical evidence [22]. In cases of abnormal serological results indicating the possibility of primary infection in pregnancy, it is advisable to perform invasive diagnostics to confirm the presence of the virus, mainly in the amniotic fluid, by identifying its genetic material by polymerase chain reaction (PCR). Diagnostic amniocentesis is the recommended method, but cordocentesis is also performed. Chorionic villus sampling is also used in some countries, but this does not eliminate the need for a diagnostic amniocentesis as the gold-standard procedure [23].

The present study is aimed at understanding the significance of ultrasound in detecting the CMV in fetuses, especially since it is a widely used and easily accessible method. This study aims to understand if ultrasound, especially targeted transvaginal neurosonography, is a satisfactory tool for the detection of findings related to cCMV infection. The review highlights the features that are diagnosed by ultrasound.

## 2. Methods

This systematic review is registered in OSF Registries (https://doi.org/10.17605/OSF.IO/6YSJ2, accessed on 19 June 2023).

The scoping review framework of Arksey and O’Malley [24] was utilized for writing this review. There are five distinct stages in this framework which include identification of the research question, followed by identifying relevant studies, selecting the appropriate studies, extracting the data, clustering the data and reporting the results. The databases used for searching the literature include Medline, PubMed and PsycInfo, Web of Science and Scopus. The keywords used for identification of studies included “CMV infection”, “Pregnancy”, “Ultrasonography”, “Reliability”. However, these keywords gave too many references, so search terms were generated and used for identification of the reference articles. The various search terms used included “epidemiology of maternal and congenital CMV infection and pregnant women”, “pathophysiology of fetal CMV infection”, “diagnostic challenges associated with CMV detection and pregnancy”, “ultrasonography detection of CMV and pregnant women”, “CMV detection in fetus and ultrasonography”, “intracranial abnormalities in fetus and CMV infection and ultrasound” and “ultrasound-based detection of fetal CMV infections and accuracy”. A summary of various search terms used for various databases is summarized in Table 1.

### 2.1. Article Selection Process and Selection Criteria

#### 2.1.1. Identification of Articles Using the Search Terms

The total number of articles from Medline/Pubmed was 174. The total number of articles from PsycINFO were 1153. Of the 1153 articles, a total of 382 book chapters, 18 encyclopedias, and 6 duplicate articles were removed in the first step to obtain 747 articles for further screening. A total of 79 articles were obtained from the Web of Science. A total of 95 articles were obtained from Scopus. Of the 95 articles, only one article was new and all the remaining ones were duplicates. Therefore, a total of 1001 articles were considered for screening from various web sources.

#### 2.1.2. Screening of Articles

Of the 1001 articles for screening, 10 records were excluded as they were not in English, so only 991 articles were further screened, based on eligibility criteria. From the 991 articles a final number of 943 articles were excluded, based on eligibility criteria.

#### 2.1.3. Inclusion

A final number of 48 articles were included for the review.

#### 2.1.4. Inclusion Criteria

Articles that were related to ultrasonography and CMV-based infections were considered. Articles that had information on comparing ultrasonography with other methods of detection in congenital CMV infections were included in the study. References related to epidemiology of CMV and the incidence of congenital anomalies associated with CMV were included. Various articles published in the research area between 2001 and 2022 were only considered.

#### 2.1.5. Exclusion Criteria

Articles that were not in English were not considered for the review. Articles that were on general aspects associated with CMV and not pertaining to the subject were not considered. Grey literature was not considered for the study.

**Figure 1:**
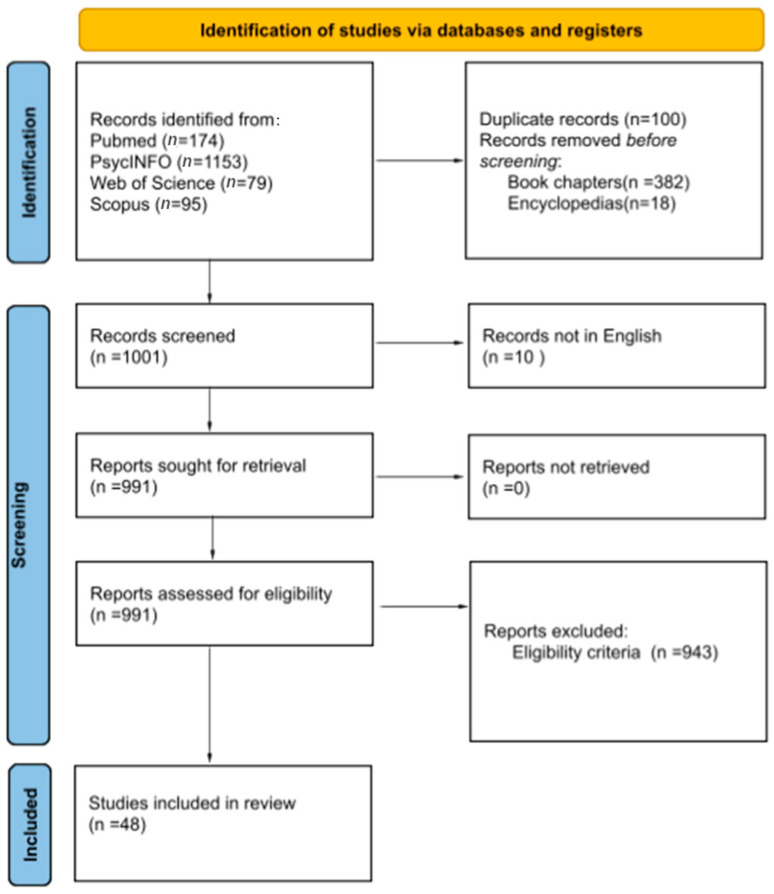
PRISMA flow sheet highlighting the selection process for finalizing the articles.

Four reviewers reviewed the whole work independently. Titles and abstracts were reviewed by one of the reviewers to identify the relevant text. Full texts of the articles and the reference list of various articles was further analyzed by two reviewers independently. Full texts of the finalized references were evaluated by two reviewers before their inclusion in the review (Figure 1).

## 3. Results

### 3.1. Sonographic Analysis

Traditional methods for fetal CMV detection include methods wherein the detection happens only after the infection of the fetus and mother has happened. Most of these methods need to collect blood or amniotic fluid and then perform the detection of CMV [25,26,27,28,29,30]. The use of non-invasive methods for monitoring infection has become a necessity of late. A summary of various studies that have used the US (ultrasound examination) for fetal anomalies is summarized in Table 2.

### 3.2. US for Fetal CMV Infection

Ultrasonography (US) for detection of congenital CMV has been reported in the literature as one of the methods to assess the growth of fetus and vertical transmission of the virus. In an observational study, fetal ultrasound was used to detect anomalies [31]. A total of 37.7% of the pregnancies screened had anomalies detected by fetal ultrasound. The US-based detection was confirmed postnatally or by performing an autopsy of the dead fetus. Infants born with CMV infection showed anomalies on the ultrasound which included neurological or hearing issues [33]. Fetal viral infections result in sonographic abnormalities which might not be very specific. However, they are indicative of intrauterine infection [37]. Sonography of the fetus is a useful indicator of the CMV infection. Ultrasonography is a very reliable indicator of the fetal and placental health status. Intrauterine infections have been characterized by increased echogenicity of internal and various provisional organs in the US [37]. In a retrospective study carried out at the hospital of the University of Montreal, of the total 84 cases around 38 cases had prenatal anomalies as detected by the US. Approximately 42% of these cases developed severe outcomes. Thus, the detection of prenatal anomalies by the US is indicative of adverse outcomes of pregnancy [51].

### 3.3. Intracranial Features Observed by US in Fetal CMV Infection

Fetal transvaginal neurosonography is the method of choice as a tool for detailed evaluation of the brain anatomy, defects, and insults. Undoubtedly, the human factor is important factor in ultrasonographic evaluation. In the situation of primary CMV infection during pregnancy, each patient should have fetal transvaginal neurosonography performed at a reference center by a person experienced in this type of examination. Only such an examination with full protocols ensures high diagnostic sensitivity. Intracranial lesions in CMV infection depend on the degree of central nervous system (CNS) involvement. They can be subtle or very exacerbated. The virus reaches the brain tissue via the bloodstream, from where it enters the cerebrospinal fluid, causing inflammation of the choroid plexuses and meningitis. Thus, the lesions appear earliest in the ventricular system and periventricular tissue, from where the virus spreads to the parenchyma [34].

A sonography of CMV-infected fetuses at a mean gestation of 27.5 weeks showed a specific pattern of infection. The three major observations associated with the fetal brain were abnormal patterns of periventricular echogenicity, ventriculomegaly and echogenic intraparenchymal foci. Abnormal patterns of sulci and gyri (malformation of cortical development) along with abnormalities in cerebellar and cisternal magna were also prominent. The presence of any two of the aforementioned features in the sonography of the fetus is indicative of CMV infection [35,50]. Several other studies have also reported many of the intracranial abnormalities, such as brain calcification and occipital horn cavitation. Also, this study observed an anechogenic cavitation in the occipital horn which was unique and not reported by other studies, for fetal CMV infection [49].

Temporal lobe involvement, manifested by temporal lobe widening, the presence of cysts and increased echogenicity of the temporal horn periventricular tissue, is a very common and suggestive pathology seen in CNS infection [34].

Many other features reported include thalamic hyperechogenicity, intracranial calcifications, intraventricular adhesions [46] and mild ventriculomegaly [52]. However, some of the rare observations from the US include lenticulostriate vasculopathy (LSV) [40,44]. It is still not clear if isolated LSV is only related to CMV infection. A periventricular echogenic halo with concomitant white matter lesions which correspond to periventriculitis [53] is described as an early sign of CMV infection. Microcephaly and white matter lesions have been reported by many studies. This is the result of the decreasing number of neurons. Early involvement of the germinal matrix may cause a decreasing pool of germinal neurons which are the source for brain parenchymal tissue. Such involvement changes are localized along the basolateral wall of the anterior horns of the lateral ventricles, medial to the head of the caudate nucleus, at the location of the GM mostly anterior to the caudothalamic groove (named the hyperechogenic caudate germinal matrix or HCGM). These lesions can be isolated (with or without LSV) or, if more diffused, they can lead to a destruction of the reproductive layer and be part of large destructive lesions involving the entire CNS [40].

A reduction in the pool of neuronal stem cells progenitors results in an inhibition of their proliferation and differentiation into neuronal and glia cells, which translates into a reduction in brain volume, visible to us as microcephaly. In addition, neurotoxic agents produced in the inflamed tissue result in a focal parenchymal area of necrosis and calcification. Additional inflammatory changes in the placenta can lead to placental insufficiency, which in turn can cause hypoperfusion, fetal hypoxia, and increased production of oxygen free radicals and contribute to some brain abnormalities such as malformation of cortical development, including polymicrogyria and white matter lesions [54].

Damage to the progenitor cell pool and disruption of both migration modes of neurons (radial and tangential neuronal migration) could be affected, and may result in anomalies of the corpus callosum (absence, hypoplasia, dysplasia), pontocerebral hypoplasia and cerebral hypoplasia [35,36]. In addition to the classic signs of vertical CMV transmission that have already been listed, ultrasound is also used to monitor further changes in the developing fetus. Transvaginal neurosonography of the fetus at 20–22 weeks of gestation with CMV infection was carried out in one such study. The presence of the halo was indicative of damage to white matter which could result in serious complications during or after pregnancy for the fetus [53]. A single case report presented for a woman with intrauterine CMV infection showed the features as listed by Malinger et al., [35]. At 31.6 weeks’ gestation, ventriculomegaly was visible by ultrasound. The CMV infection was confirmed by RT-PCR (reverse transcription polymerase chain reaction) and MRI also confirmed the parenchymal changes. Parenchymal cystic change was further confirmed by ultrasound a day before delivery of the child [55]. Detection of ocular changes associated with CMV infection, other than microphthalmia and cataracts, is beyond the capabilities of targeted fetal neurosonography. Optic nerve atrophy or hypoplasia is also a rare complication described following congenital infection, which is hardly possible to detect by targeted fetal neurosonography [32].

### 3.4. Extracranial Features Associated with Fetal CMV Infection

The extracranial features associated with infection include hyperechogenic bowel, cardiomegaly, hepatosplenomegaly and pericardial effusion [46]. Hyperechogenic bowel is the most common finding reported by most of the studies [38,41,47,49,50,51,52]. CMV infection of the fetus when diagnosed with US also shows relatively rare features. In one such study, the US of a woman at 29 weeks of gestation showed micrognathia, flat nose, cleft lip and palate. The study for the first time reports unusual observations, which support CMV infection resulting in damage to oral and facial development [42]. However, there is no clear link to the occurrence of strictly anatomical defects in the fetus with CMV infection as a single cause of such anomalies. Persistent pulmonary hypertension of the newborn (PPHN) is another rare manifestation of cCMV infection [43]. Anomalies of hyperechogenic bowel and oligohydramnios are detected as main features in mothers with cerebral involvement during CMV infection of the fetus [47]. Fetal hyperechogenic bowel detection by US is one of the major features that is associated with fetal abnormalities. It could be mainly associated with a variety of factors, including congenital infections. CMV infections account for 2.2% of the total echogenic bowel cases detected by US [56]. In pregnancies with intrauterine CMV infection, placentomegaly was observed significantly more often. Calcification as an expression of tissue necrosis was observed mainly in the fetal liver [57]. Fetuses with congenital infection are more likely to present growth abnormalities in the form of FGR or SGA [39,57,58]. 

## 4. Discussion

The diagnosis of vertical infection of the fetus with CMV poses a great challenge, and thus is associated with its problems [45].

Undoubtedly, an important factor in the discussion of the role of the US here is the distinction between the two types of US examination. Screening the population using US examination can detect certain changes which in further diagnostic stages will allow the establishment of the etiology of the observed changes, in this case an intrauterine CMV infection. A dedicated (targeted) US examination in specific situations, e.g., examination in a group of patients with primary infection in pregnancy either before or after performing invasive diagnostics confirming the presence of intrauterine infection, and the serial monitoring of patients with already-confirmed intrauterine infection for the appearance of specific symptoms in a follow-up US examination is obligatory. The above-mentioned group of patients, besides detailed fetal scanning, should be offered targeted transvaginal fetal neurosonography by reference centers with extensive experience in monitoring and detecting signs of fetal disease, especially brain involvement.

A greater risk of postnatal illness exists in fetuses with congenital CMV infection and aberrant sonographic evidence. Symptomatic congenital infection in fetuses subjected to maternal CMV infection when the infection condition is unknown can only be correctly determine by ultrasonography in one third of cases. Contrarily, a normal fetal anatomical assessment may help assure patients who are vulnerable to fetal symptomatic infection [52]. When a primary infection is found in early pregnancy, ultrasound becomes the only source of information for its structure and organ involvement. Although ultrasonography is not the most reliable way to detect fetal CMV infection, it can be a helpful tool in assessing the possibility of postnatal CMV disease. Parental counseling can be tailored to the condition of the fetal infection after invasive prenatal testing; supporting evidence that suggests ultrasonographic anomalies associated with confirmed in utero fetal infection may increase the chance of poor neonatal outcomes. In fact, when the results show fetal infection, the PPV (positive predictive value) of ultrasonography rises twofold. It is challenging to identify ultrasonography findings that are important in the pathogenesis of CMV infection amongst some of the anomalies seen in infected fetuses [41]. The most typical aberrant signs include ventriculomegaly [35,50] and hyperechogenic bowel [38,41,47,49,50,51], though these can also be found in uninfected fetuses. About 5% of fetal ventriculomegaly cases are caused by an infectious agent.

Following the confirmation of CMV in amniotic fluid, ultrasound examinations are much more targeted, and greater effort is made to identify any fetal harm. It is well established that ultrasonography has limits in identifying risk factors for poor fetal outcomes, and that a variety of factors can affect how often prenatal infections are detected. There are significant differences in how well fetal ultrasound examinations function, depending on the severity of infection. Only babies with severe CMV infection will exhibit evident ultrasound abnormalities, while more subtle ultrasound characteristics are probably undetected unless a targeted or fetal neurosonography is carried out by an expert [44,59,60]. In a study by Buca et al. [48] reporting the outcomes of fetuses with congenital cytomegalovirus (cCMV) infection and a normal ultrasound at the time of their diagnosis, it was discovered that the threat of poor postnatal consequences is lower in fetuses with congenital CMV infection displaying no oddities in the prenatal image analysis than that which was revealed in earlier reported papers, not taking into account the importance of antenatal imaging evaluation. Approximately 4% of patients had additional defects discovered only at follow-up ultrasounds, underlining the importance of a continuous assessment of the impacted fetuses throughout pregnancy [46]. Most significantly, symptomatic infection and aberrant neurological developmental outcomes occurred in 1.7% and 3.2%, correspondingly, in fetuses with normal prenatal imaging verified at birth, whereas auditory issues occurred 6.5% of the time. The extremely limited quantity of all kinds of instances and absence of comparability of the results in the initial studies had an impact on sub-analyses based on the trimester at the time of infection [46]. First-trimester infection increased the chance of further defects at subsequent ultrasound scans, poor neurodevelopmental outcomes, and auditory issues in fetuses.

Even though ultrasonographic findings in a pregnant woman with a primary CMV infection strongly suggest fetal infection, they are not fully diagnostic strictly for cCMV, because they share characteristics with some other fetal disorders. Fewer than half of the affected fetuses also exhibit abnormalities on the US scan [49]. However, novel ultrasound criteria for the detection of infected fetuses for danger of signs after delivery have not yet been developed. Some modest ultrasound indicators have been proposed to detect at-risk pregnancies. Despite having low sensitivity for prenatal diagnosis, ultrasound imaging can be helpful in predicting the prognosis of fetal infection in some cases [61]. 

## 5. Conclusions

Ultrasound is a readily available method, used widely in modern obstetric medicine, and has been very useful in detecting the fetal brain and other organ-related abnormalities, especially if performed by an expert neurosonographer with a full protocol neurosographic examination. However, abnormalities associated with fetuses in ultrasound could be due to any of the fetal viral infections and not be specific strictly to CMV alone. Thus, the establishment of CMV infection alone with sonography is not possible, and needs an additional molecular tool for confirmation. As CMV infection constitutes the most prevalent viral infection across the world, it should be considered first in differential diagnosis. Temporal lobe involvement and occipital horn cavitation with increased periventricular echogenicity are very indicative of CMV infection of the fetus. However, sonographic analysis at regular intervals of gestation could be a reliable indicator of fetal changes that could help in predicting the antenatal outcome and thus could be suggestive to the doctors of the course of therapy to be undertaken. The review definitively answers the objective of our study, that the US is a reliable indicator of fetal CMV-based infections, but alone can be not enough to detect mild symptoms of CMV infection, especially in the fetal brain.

## Figures and Tables

**Table 1:** Summary of the various search terms used in different web bases.

Data Base	Search Terms
Medline/Pubmed	Epidemiology of maternal and congenital CMV infection and pregnant women (Title/Abstract), pathophysiology of fetal CMV infection (Title/Abstract), diagnostic challenges associated with CMV detection and pregnancy (Title/Abstract), ultrasonography detection of CMV and pregnant women (Title/Abstract), CMV detection in fetus and ultrasonography (Title/Abstract), intracranial abnormalities in fetus and CMV infection and ultrasound (Title/Abstract), ultrasound-based detection of fetal CMV infections and accuracy (Title/Abstract)
PsycInfo	CMV detection in fetus and ultrasonography (Title/Abstract), intracranial abnormalities in fetus and CMV infection and ultrasound (Title/Abstract)
Web of Science	Epidemiology of maternal and congenital CMV infection and pregnant women (Title/Abstract), pathophysiology of fetal CMV infection (Title/Abstract), diagnostic challenges associated with CMV detection and pregnancy (Title/Abstract), ultrasonography detection of CMV and pregnant women (Title/Abstract), CMV detection in fetus and ultrasonography (Title/Abstract), intracranial abnormalities in fetus and CMV infection and ultrasound (Title/Abstract), ultrasound-based detection of fetal CMV infections and accuracy (Title/Abstract)
Scopus	Epidemiology of maternal and congenital CMV infection and pregnant women (Title/Abstract), pathophysiology of fetal CMV infection (Title/Abstract), diagnostic challenges associated with CMV detection and pregnancy (Title/Abstract), ultrasonography detection of CMV and pregnant women (Title/Abstract), CMV detection in fetus and ultrasonography (Title/Abstract), intracranial abnormalities in fetus and CMV infection and ultrasound (Title/Abstract), ultrasound-based detection of fetal CMV infections and accuracy (Title/Abstract)

**Table 2:** Summary of various studies utilizing US-based anomalies in fetal CMV infections.

Author	Year	Study Group	Methodology	CMV Signs in US	Outcomes
De Catte et al. [30]	2012	Review	US-based screening for fetal anomalies.	Microcephaly, cortical malformations and intraparenchymal cysts.	The three signs in US are associated with poor outcome of delivery.
Leyder et al. [31]	2016	A total of 69 fetuses with proven vertical transmission of pregnancy. Of these, 64 were singleton and 3 were twin pregnancies.	Prospective observational study from 1996 to 2012.	Echogenic bowel with periventricular echogenic halo and inclusions in lung, liver, pancreas and kidney.	Women who continued their pregnancy had infants with hearing and neurological outcomes.
Lipitz et al. [32]	2010	A total of 38 singleton pregnancies with vertical transmission of CMV.	Prospective cohort study of singleton pregnancy between 2004 and 2009.	Echogenic bowel with periventricular cysts in caudate nucleus.	Normal MRI and US show a normal pregnancy. However, normal MRI with anomalies in US have neurological and hearing outcomes.
Degani et al. [33]	2006	A total of 16 infected fetuses in one study, 8 infected fetuses in another study and 189 CMV-infected pregnancies in the third study.	Analysis of fetal anomalies in viral infections.	Ventriculomegaly, intracranial and hepatic calcifications.	Abnormalities in US need thorough fetal evaluation.
Malinger et al. [34]	2003	Eight pregnant women with confirmed CMV infection.	Transadbominal and transvaginal sonograms for patterns of infection in infected fetus.	Periventricular echogenicity was seen in all fetuses. Ventriculomegaly, intraparenchymal foci, abnormal patterns of gyre and sulci, anomalies of corpus callosum, abnormalities of cerebral and cisternal magna, and vasculopathy of striatal artery showed differences in transabdominal sonography which were further confirmed by transvaginal sonography.	Presence of any of the two symptoms in sonography needs a proper evaluation of fetus.
O’Sullivan et al. [35]	2017	Single pregnancy	Case report with women with medical history of epilepsy.	Echogenic bowel, bilateral ventriculomegaly, periventricular hyperechogenicity.	US detects abnormalities in pregnancy and the risk associated with loss of fetus.
Moinuddin et al. [36]	2003	Single pregnancy with CMV infection.	Case report with 20-year-old woman showing isolated fetal ascites on US.	Cerebral ventriculomegaly with parenchymal cystic changes.	US, along with MRI, is very useful in fetal abnormalities.
Minsart et al. [37]	2020	A total of 84 mother–child pairs were screened at Montreal between 2003 and 2017.	Retrospective study to confirm congenital CMV in absence of screening.	Extracerebral anomalies were most common, and few fetuses had changes in neurological tissues.	Cerebral palsy, severe cognitive impairment, bilateral hearing loss or neonatal death were observed in fetus which had severe symptoms associated with CMV infection.
Nigro et al. [38]	2002	Single pregnancy with confirmed CMV infection.	US of fetus along with nested-PCR and CMV antibody detection.	Intraventricular hemorrhage along with oligohydramnios and hyperechogenic bowel.	Intracranial hemorrhage shown for first time in fetus due to CMV infection.
Picone et al. [39]	2008	A total of 38 pregnancies with congenital CMV infection.	Comparative study of pregnancies for anomaly detection by MRI and US.	Extracerebral features and cerebral features were detected in Groups 2 and 3.	MRI showed additional features when compared to the US. Also, where US was negative, CMV features were detected on MRI.
Simonazzi et al. [40]	2010	A total of 218 patients with fetal CMV infections.	Transvaginal US at mid gestation.	Periventricular echogenic halo with well-defined borders. It was associated with white matter lesions.	Periventricular echogenic halo mostly associated with white matter lesions, and patients opted for termination of pregnancy.
Weichert et al. [41]	2010	Single case of 29-weeks gestation.	US of CMV-infected fetus at 29 weeks of gestation.	Micrognathia, single-cleft lip, ventriculomegaly, brain structure anomalies and oligohydramnios.	First report of micrognathia and cleft lip in humans.
Babu et al. [42]	2018	Single case of congenital CMV infection.	Fulminant congenital CMV and antiviral treatment. Observations post delivery of child.	Multiple ventricular calcifications and intraventricular hemorrhages.	Persistent pulmonary hypertension of newborn (PPHN) is a rare manifestation of congenital CMV infection.
D’Amico et al. [43]	2021	A total of 12971 fetuses were included in the study.	Isolated echogenic bowel on antenatal ultrasound was assessed in the study.	A total of 2.2% of cases with isolated echogenic bowels were associated with CMV infection.	The fetuses with echogenic bowel were at increased risk of adverse outcomes.
Birnbaum et al. [44]	2021	A total of 22 infected fetuses. Group A included 18 fetuses and Group B included 4 fetuses.	Retrospective neurosonography analysis and outcome correlation.	Focal changes in germinal matrix (GM) are observed often with coexistence with lenticulostriate vasculopathy (LSV) late in pregnancy.	The changes in US could indicated brain damage in fetus.
Birnbaum et al. [45]	2017	A total of 81 CMV-infected fetuses.	Comparison of brain MRI and neurosonography for CMV-infected fetus.	Ventriculomegaly, periventricular or porencephalic cysts, periventricular increased echogenicity, occipital horn cavitation, brainatrophy/destruction, cerebellar and callosal abnormalities.	Inconclusive results with MRI are more than with US. Adding MRI to US would give more conclusive results.
Guerra et al. [46]	2008	A total of 650 fetuses from mothers infected with CMV.	Effectiveness of ultrasound in prediction of antenatal CMV.	Choroid plexus cysts, mild unilateral pyelectasis, hyperechogenic bowel, ventriculomegaly and enlargement of cisterna magna.	US-based prediction of congenital CMV is seen only in one third of fetuses.
Imafuku et al. [47]	2020	A total of 4380 pregnancies were screened for CMV infection.	Prospective cohort study.	Ventriculomegaly, intracranial calcification, hyperechogenic bowel, microcephaly, hepatosplenomegaly and ascites.	US fetal abnormalities in conjunction with nucleic acid screening for CMV is ideal method for confirmation.
Buca et al. [48]	2021	A total of 2603 fetuses with congenital CMV infection.	US for detection of outcome of fetus with congenital CMV infection.	Intra-CNS and extra-CNS anomalies.	MRI detected anomalies in CMV infection in those scans where ultrasound was normal.
Dogan et al. [49]	2011	Fetal CMV infection	Study on eight fetuses with confirmed CMV infection for intracranial and extracranial abnormalities.	Increased periventricular echogenicity, ventriculomegaly, intracranial calcifications, intraventricular adhesions, thalamic hyperechogenicity, enlarged mega cisterna magna, cerebellar cyst. Extracranial included hyperechogenic bowel, cardiomegaly, hepatosplenomegaly and pericardial effusion.	US observations of intracranial abnormalities are prominent feature of CMV infection.
Picone et al. [50]	2014	Fetal CMV infection	Retrospective evaluation of 69 confirmed CMV-infected patients between 2004 and 2013.	Extracerebral included hyperechogenic bowel and intrauterine growth retardation. Intracerebral included brain calcification and occipital horn cavity. Anechogenic cavity at extreme end of occipital horn.	US findings are not specific to CMV infection. The anechogenic cavity is a novel observation of the study.

US—ultrasound examination, MRI—magnetic resonance, PCR—polymerase chain reaction, LSV- lenticulostriate vasculopathy.

## Data Availability

Not applicable.
